# Perioperative cardiovascular complications versus perioperative bleeding in consecutive patients with known cardiac disease undergoing non-cardiac surgery. Focus on antithrombotic medication. The PRAGUE-14 registry

**DOI:** 10.1007/s12471-014-0575-3

**Published:** 2014-08-14

**Authors:** P. Widimský, Z. Moťovská, L. Havlůj, M. Ondráková, R. Bartoška, L. Bittner, L. Dušek, V. Džupa, J. Knot, M. Krbec, L. Mencl, J. Pachl, R. Grill, P. Haninec, P. Waldauf, R. Gürlich

**Affiliations:** 1 Cardiocenter, University Hospital Kralovske Vinohrady Prague and Charles University Prague, Srobarova 50, Prague 10, Czech Republic; 2 Institute of Biostatistics and Analyses, Masaryk University, Brno, Czech Republic; 3 Cardiocenter, University Hospital Kralovske Vinohrady, Srobarova 50, Prague 10, Czech Republic

**Keywords:** Non-cardiac surgery, Perioperative ischemia, Perioperative bleeding, Thrombosis, Antithrombotic therapy, Aspirin, Warfarin, Thienopyridines

## Abstract

**Background:**

Interruption of antithrombotic treatment before surgery may prevent bleeding, but at the price of increasing cardiovascular complications. This prospective study analysed the impact of antithrombotic therapy interruption on outcomes in non-selected surgical patients with known cardiovascular disease (CVD).

**Methods:**

All 1200 consecutive patients (age 74.2 ± 10.2 years) undergoing major non-cardiac surgery (37.4 % acute, 61.4 % elective) during a period of 2.5 years while having at least one CVD were enrolled. Details on medication, bleeding, cardiovascular complications and cause of death were registered.

**Results:**

In-hospital mortality was 3.9 % (versus 0.9 % mortality among 17,740 patients without CVD). Cardiovascular complications occurred in 91 (7.6 %) patients (with 37.4 % case fatality). Perioperative bleeding occurred in 160 (13.3 %) patients and was fatal in 2 (1.2 % case fatality). Multivariate analysis revealed age, preoperative anaemia, history of chronic heart failure, acute surgery and general anaesthesia predictive of cardiovascular complications. For bleeding complications multivariate analysis found warfarin use in the last 3 days, history of hypertension and general anaesthesia as independent predictive factors. Aspirin interruption before surgery was not predictive for either cardiovascular or for bleeding complications.

**Conclusions:**

Perioperative cardiovascular complications in these high-risk elderly all-comer surgical patients with known cardiovascular disease are relatively rare, but once they occur, the case fatality is high. Perioperative bleeding complications are more frequent, but their case fatality is extremely low. Patterns of interruption of chronic aspirin therapy before major non-cardiac surgery are not predictive for perioperative complications (neither cardiovascular, nor bleeding). Simple baseline clinical factors are better predictors of outcomes than antithrombotic drug interruption patterns.

## Background

Interruption of antithrombotic therapy may be detrimental in some patients. Meta-analysis of six studies on aspirin adherence revealed that aspirin non-adherence/withdrawal was associated with a threefold higher risk of major adverse cardiac events [1]. Non-cardiac surgery is frequently accompanied by interruption of antithrombotic therapy and thus increases the risk of stent thrombosis, myocardial ischaemia/infarction, and death, especially soon after stent placement. The risk of surgical haemorrhage is increased 20 % by aspirin or clopidogrel alone and 50 % by dual antiplatelet therapy. Therefore, some authors recommend to continue antiplatelet therapy throughout the perioperative period except when the risk of bleeding outweighs the risk of acute stent thrombosis, while others suggest 5–10 days interruption prior to surgery [2].

In moderate to high risk patients receiving acetylsalicylic acid (ASA) and requiring non-cardiac surgery, the American College of Clinical Pharmacy (ACCP) recommends ASA to be continued around the time of surgery. Perioperative antithrombotic management should be based on individual risk assessment for thrombotic versus bleeding risk [3]. In patients with semi-urgent surgery, the decision to prematurely stop antiplatelet agents has to be taken after multidisciplinary consultation, evaluating the individual thrombotic and bleeding risk. Urgently needed surgery has to take place under full antiplatelet therapy despite the increased bleeding risk. A multidisciplinary approach for optimal antithrombotic and haemostatic patient management is mandatory in this situation [4]. Multiple studies have reported an independent association between postoperative myocardial ischaemia and major adverse cardiac events (MACE) and mortality, in both the short and the long term [5].

Surgeons usually recommend to stop all antithrombotic medication 1 week before surgery to prevent perioperative bleeding. Such a strategy may increase the risk of perioperative cardiovascular (frequently thrombotic / ischaemic) complications. This study was designed to collect data from all consecutive (non-selected) patients with known cardiovascular disease undergoing acute or elective major non-cardiac surgery in order to analyse the bleeding / thrombotic risk balance with focus on antithrombotic therapy.

## Methods and patients

The study protocol was approved by the Ethics Committee of the University Hospital Kralovske Vinohrady and by the Czech Ministry of Health (IGA). The study is registered on www.ClinicalTrials.gov under the identifier NCT01897220.

All departments performing major non-cardiac surgery in a large tertiary university hospital participated: general surgery (43.3 % patients), trauma and orthopaedic surgery (39.9 %), urology (10.5 %), neurosurgery (5.5 %) and anaesthesiology (0.8 %). The study was designed and coordinated by the department of cardiology in the same hospital. All 1200 consecutive patients undergoing non-cardiac surgery during the study period, while having known cardiovascular disease, were enrolled (6.3 % of 18,951 patients undergoing non-cardiac surgery during the study period 2011–2013).

The cardiovascular diagnoses at baseline (which formed the only entry criteria for the study) were coronary artery disease (*n* = 820), atrial fibrillation (*n* = 369), valvular disease (*n* = 176), prior stroke (*n* = 127), presence of a prosthetic valve (*n* = 72), congestive heart failure (*n* = 48), prior venous thromboembolism (*n* = 40) and cardiomyopathy (*n* = 23). Patients could have more than one (e.g. coronary artery disease and atrial fibrillation).

Acute (urgent or emergent, i.e. done during an acute unscheduled hospital admission) surgery was performed in 37.4 % of patients, elective (planned) surgery in the remaining 61.4 %, while in 1.2 % this was unspecified. General anaesthesia was used in 64.4 %, another type of anaesthesia (epidural, subarachnoid) in 35.6 %.

The decision to stop antithrombotic medication preoperatively was left to the discretion of attending physicians (usually a cardiologist or internist in collaboration with the anaesthesiologist or surgeon). No specific recommendations were prescribed by the study protocol.

The following data were recorded during the hospital stay: ECG, medication (prior, during and after surgery), blood counts, INR, creatinine, type of surgery, type of anaesthesia, perioperative bleeding, perioperative cardiovascular complications (myocardial infarction, clinically manifest pulmonary embolism or deep venous thrombosis, acute limb ischaemia, acute stroke, new onset or recurrent heart failure) and the cause of death. Perioperative bleeding was classified as (A) fatal, (B) postoperative serious bleeding requiring surgical revision, (C) postoperative serious bleeding requiring transfusions but not revision, (D) intraoperative serious bleeding complicating the procedure and (E) intraoperative bleeding greater than usual prolonging the procedure. This classification has significant similarities (Table 1) with the classification of the International Society on Thrombosis and Haemostasis (ISTH) [6]. We decided on a simple original classification of bleeding, also in the light of certain criticism of the ISTH classification [7].

**Table 1:** Perioperative bleeding complications

Type of bleeding (PRAGUE-14 classification)	Number of patients	Type of bleeding (ISTH classification)	Number of patients
Fatal (type A)	2	Fatal (class 1)	2
Postoperative serious requiring surgical revision (type B)	16	Surgical requiring second intervention (class 4)	16
Postoperative serious requiring transfusions but not revision (type C)	25	Large surgical without second intervention (class 5)	35
Intraoperative serious complicating surgery (type D)	13		
Intraoperative greater than usual, prolonging surgery (type E)	103		
		Extrasurgical leading to transfusions (class 3)	2
		Bleeding in critical area or organ (class 2)	1

### Statistical analyses

Electronic case report forms were prepared with software Oracle XE 10 G, data mining was done with Rapidminer 5.3, and data were exported to Microsoft Excel 2010. Statistical analyses were performed using Statistica 8 Statsoft, SPSS 20.0.1 (IBM Corporation, 2011) and RStudio 0.96.331 (R Core Team, 2012).

Standard descriptive statistics were applied in the analysis; categorical variables were described using absolute and relative frequencies, continuous variables using mean and standard deviation. Statistical significance of association between categorised parameters was assessed using Fisher’s exact test for both 2 × 2 and r × c contingency tables; for categorical variables Pearson’s parametric coefficient of correlation was used to assess the relationship among continuous parameters [8]. Non-parametric Mann–Whitney was applied to test differences in continuous variables among groups of patients. We employed logistic regression models to assess the association between potential predictors and selected binary coded endpoints (perioperative ischaemia, perioperative bleeding and hospital mortality). Both univariate and multivariate regression models were applied, resulting in age-adjusted and multivariate-adjusted estimates of odds ratio with corresponding 95 % confidence limits. All factors reaching *p* value <0.1 in the univariate models and their properly coded interaction terms (if significant) entered the multivariate models as initial pool of independent variables. A stepwise backward procedure was applied to filter the final set of mutually independent predictors providing the best model. The models used maximum likelihood estimation directly comparing the likelihood *L0* for the null model where all slope parameters are zero, with the likelihood *L1* of the fitted model. Significance of regression coefficients was tested with the help of the Wald statistic, which is based on the asymptotic normality property of maximum likelihood estimates (tested against Chi-square distribution). All statistical tests were two-sided, and a value α <0.05 was considered to be a threshold for statistical significance in all the comparisons made.

## Results

### Patient characteristics

The patients’ baseline characteristics are shown in Table 2. The baseline chronic antithrombotic medication included ASA (53.2 %), warfarin ± ASA (24.6 %), ASA + clopidogrel (3.7 %), clopidogrel alone (1.7 %), dabigatran (0.3 %) and no antithrombotic agents (16.5 %). Other chronic medication included beta-blockers (61.4 %), angiotensin-converting enzyme inhibitors (54.1 %), statins (29.8 %), diuretics (40.2 %), calcium channel blockers (19 %) and nitrates (8.1 %).

**Table 2:** Baseline characteristics of study patients per complication type

Parameter	Uncomplicated patients	Patients with cardiovascular complications	Patients with bleeding complications	Patients with both types of complications
N =	973	67	136	24
Mean age	73.9 ± 10.2	78.9 ± 11.5	73.4 ± 8.8	77.5 ± 9.8
Female sex	434 (44.6 %)	31 (46.3 %)	52 (38.2 %)	10 (41.7 %)
Mean body weight	79.1 ± 16.2	76.7 ± 19.9	79.2 ± 14.0	74.0 ± 15.4
Diabetes mellitus	291 (29.9 %)	24 (35.8 %)	46 (33.8 %)	10 (41.7 %)
Hypertension	758 (77.9 %)	48 (71.6 %)	111 (81.6 %)	17 (70.8 %)
Chronic kidney disease	110 (11.3 %)	5 (7.5 %)	17 (12.5 %)	3 (12.5 %)
Chronic liver disease	44 (4.5 %)	2 (3.0 %)	5 (3.7 %)	3 (12.5 %)
Chronic pulmonary disease	121 (12.4 %)	13 (19.4 %)	15 (11.0 %)	6 (25.0 %)
Current tumour	152 (15.6 %)	11 (16.4 %)	20 (14.7 %)	4 (16.7 %)
Current haematological disease	30 (3.1 %)	1 (1.5 %)	6 (4.4 %)	1 (4.2 %)
Presence of vascular (coronary or peripheral) stent	195 (20.0 %)	12 (17.9 %)	36 (26.5 %)	9 (37.5 %)
History of any previous bleeding requiring treatment	9 (0.8 %)	1 (1.2 %)	0 (0 %)	0 (0 %)

### Perioperative complications

Perioperative cardiovascular complications occurred in 91 (7.6 %) patients: acute or worsening heart failure (*n* = 46), acute myocardial infarction (*n* = 24), venous thromboembolism (*n* = 15), acute limb ischaemia (*n* = 11) and acute stroke (*n* = 3). Multivariate analysis revealed the following independent risk factors for perioperative cardiovascular complications: age, acute surgery (versus elective), preoperative anaemia, history of previous PCI, history of chronic heart failure, general anaesthesia (versus other types of anaesthesia). For details see Table 3.

**Table 3:** Univariate (age-adjusted) and multivariate-adjusted estimates of odds ratios of predictors associated with perioperative cardiovascular complications as risk end-point

Predictors (Risk and reference categories)		Univariate age-adjusted model			Multivariate model		
		OR	95 % IC	p^*^	OR	95 % IC	p^*^
Baseline characteristics							
Age (continuous)	Adjusting factor	1.05	1.03–1.08	<0.001	1.04	1.02–1.08	<0.001
Haemoglobin: below normal	Reference: norm	2.26	1.45–3.52	<0.001	1.91	1.21–3.03	0.005
Platelets: above norm	Reference: norm	2.55	1.19–5.47	0.016			
Creatinine: above norm	Reference: norm	1.81	1.16–2.82	0.009			
Prior PCI: yes	Reference: no	2.25	1.35–3.78	0.002	1.91	1.09–3.34	0.023
Chronic heart failure: yes	Reference: no	3.12	1.48–6.57	0.003	2.80	1.26–6.22	0.011
Hypertension: yes	Reference: no	0.61	0.37–0.99	0.043			
Current smoking: yes	Reference: no	2.05	1.03–4.11	0.042			
Treatment							
ASA interrupted ≤ 7 days	Reference: > 7 days or no ASA	1.32	0.85–2.06	n.s.			
	Reference: > 7 days	1.87	0.84–4.13	n.s.			
ASA interrupted ≤ 3 days	Reference: > 3 days or no ASA	1.71	1.04–2.78	0.031			
	Reference: > 3 days	1.68	0.79–3.62	n.s.			
Clopidogrel/Ticlopidin interrupted ≤ 3 days	Reference: > 3 days	1.49	0.29–7.54	n.s.			
Warfarin interrupted ≤ 3 days	Reference: > 3 days or no Warfarin	2.61	1.16–5.86	0.020			
	Reference: > 3 days	2.94	0.93–9.23	n.s.			
Surgery characteristics							
Type of operation: acute	Reference: elective	3.94	2.38–6.54	<0.001	4.00	2.41–6.66	<0.001
General anaesthesia: yes	Reference: no	1.99	1.22–3.28	0.006	2.22	1.31–3.78	0.003
Blood loss greater than usual: yes	Reference: no	2.08	1.21–3.59	0.008			

p^*^ Wald’s test

Perioperative bleeding occurred in 160 patients (13.3 %), 24 of them also had a cardiovascular complication. Bleeding complications are shown in Table 1. Mean perioperative blood loss was 149 ± 375 ml (median 0, IQR 0–200). Red blood cell transfusions were used in 12.5 %, frozen plasma in 5 % patients, thrombocytes in 0.2 %. Multivariate analysis found three independent predictors for bleeding complications: warfarin use in the last 3 days, history of hypertension, and general anaesthesia. Surprisingly, the length of aspirin interruption before surgery was not predictive for either cardiovascular or for bleeding complications (Table 4).

**Table 4:** Univariate (age-adjusted) and multivariate-adjusted estimates of odds ratios of potential predictors associated with perioperative bleeding as risk end-point

Predictors (risk and reference categories)		Univariate model			Multivariate model		
		OR	95 % IC	p^*^	OR	95 % IC	p^*^
History							
Hypertension: yes	Reference: no	1.54	0.99–2.41	0.045	1.61	1.03–2.53	0.037
Predictors (risk and reference categories)		Univariate age-adjusted model			Multivariate model		
		OR	95 % IC	p^*^	OR	95 % IC	p^*^
Antithrombotic treatment							
ASA therapy in the last 30 days: yes	Reference: no	0.72	0.52–1.03	n.s.			
Number of days before surgery when ASA was interrupted: continuous		0.99	0.96–1.02	n.s.			
Only for acute operation		1.01	0.92–1.10	n.s.			
Only for elective operation		0.99	0.94–1.03	n.s.			
ASA 7 days: interrupted ≤7 days	Reference: >7 days or ASA not applied	0.74	0.51–1.07	n.s.			
	Reference: >7 days	0.97	0.57–1.62	n.s.			
ASA 3 days: interrupted ≤3 days	Reference: >3 days or ASA not applied	0.81	0.51–1.27	n.s.			
	Reference: >3 days	1.05	0.60–1.85	n.s.			
Number of days before surgery when Clopidogrel/Ticlopidin was interrupted: continuous		0.88	0.72–1.08	n.s.			
Warfarin therapy in the last 30 days: yes	Reference: no	1.56	1.09–2.24	0.017			
Number of days before surgery when warfarin was interrupted: continuous		0.97	0.92–1.02	n.s.			
Only for acute operation		0.89	0.77–1.04	n.s.			
Only for elective operation		0.99	0.94–1.06	n.s.			
Warfarin 3 days: interrupted ≤3 days	Reference: >3 days or Warfarin not applied	3.12	1.62–6.01	0.002	3.11	1.61–6.02	0.001
	Reference: >3 days	1.67	0.62–4.50	n.s.			
Last INR: continuous		1.11	0.81–1.50	n.s.			
Last INR >1.2	INR ≤1.2	0.89	0.47–1.69	n.s.			
Surgery characteristics							
Type of operation: acute	Reference: elective	1.27	0.91–1.78	n.s.			
General anaesthesia: yes	Reference: no	1.47	1.02–2.11	0.037	1.47	1.01–2.14	0.040

^*^Wald’s test

### In-hospital mortality

Forty-seven patients died during the hospital stay (in-hospital mortality 3.9 %). This is 4.3-times higher than the 0.9 % mortality among the remaining 17,740 patients without heart disease who underwent major surgery during the study period. The cause of death was heart failure in 20 patients, pulmonary embolism in 7, myocardial infarction in 3, sudden death in 2, acute stroke in 2, and bleeding caused death in 2 patients. Another cause of death was found in 7 patients. Thirty-four of the 91 patients with perioperative cardiovascular complications died (case fatality of cardiac patients with perioperative cardiovascular complications 37.4 %). Multivariate analysis revealed the following predictors of in-hospital mortality: age, preoperative anaemia, history of chronic heart failure, acute surgery and general anaesthesia (Table 5).

**Table 5:** Univariate (age-adjusted) and multivariate-adjusted estimates of odds ratios of potential predictors associated with hospital mortality as risk endpoint

Predictors (Risk and reference categories)		Univariate age-adjusted model			Multivariate model		
		OR	95 % IC	p^*^	OR	95 % IC	p^*^
Basic characteristics							
Age (continuous)	Adjusting factor	1.05	1.03–1.08	<0.001	1.05	1.02–1.09	0.004
Haemoglobin: under norm	Reference: norm	2.89	1.57–5.33	0.001	2.57	1.36–4.87	0.004
Chronic heart failure: yes	Reference: no	4.22	1.76–10.10	0.001	4.25	1.69–10.68	0.002
Type of surgery: acute	Reference: elective	2.81	1.44–5.50	0.003	3.00	1.53–5.88	0.001
General anaesthesia: yes	Reference: no	3.61	1.68–7.74	0.001	4.14	1.85–9.24	0.001

^*^Wald’s test

### Antithrombotic medication

Antithrombotic medication was stopped at a median of 7 (aspirin, IQR 2–10), 4 (thienopyridine, IQR 1–8) and 8 (warfarin, IQR 5–10) days prior to surgery. Perioperative preventive low-dose anticoagulation (most frequently enoxaparin) was used in 94.8 % of patients.

### Aspirin interruption

Among the patients with perioperative cardiovascular complications, aspirin was stopped a median of 2.5 days (IQR 0 – 6) before surgery (Table 6) while in patients without cardiovascular complications aspirin was stopped a median of 7 days (IQR 2 – 10) before surgery (*p* < 0.001). Among patients with perioperative bleeding, aspirin was stopped a median of 7 days (IQR 2 – 12) before surgery while in patients without bleeding aspirin was stopped a median of 7 days (IQR 2 – 10) before surgery (n.s.). Surprisingly, the median time of preoperative aspirin interruption was 7 days (IQR 2–12) among patients with bleeding complications versus 2.5 days (IQR 0–6) in those with cardiovascular complications (*p* = 0.001).

**Table 6:** Length of antithrombotic drugs interruption in subgroups per complication type

	How many days before surgery the drug was interrupted in subgroups per complication types								
		N=	Mean	SD	Median	Percentile 25	Percentile 75	Min	Max
ASA	No complications	563	7.6	7.9	7	2	10	0	90
	CV complications	38	4.1	4.8	2.5	0	6	0	18
	Bleeding	67	10.3	13.8	7	2	12	0	90
	Both types of complications	10	4.4	6.6	1	0	6	0	20
Clopidogrel	No complications	44	6.1	6.1	5	1	8	0	24
	CV complications	6	6.5	7.9	3	0	15	0	18
	Bleeding	11	3.5	4.3	1	0	7	0	12
	Both types of complications	1	11		11	11	11	11	11
Warfarin	No complications	191	9.4	7.9	8	5	11	0	60
	CV complications	16	8.4	7.3	7.5	2.5	10	0	23
	Bleeding	40	9	10.1	7	3	9.5	0	46
	Both types of complications	5	5	6	3	1	6	0	15

### Warfarin interruption

There was no significant difference (*p* = 0.80) in the median length of warfarin interruption between patients with perioperative bleeding (7 days, IQR 3 – 9.5) versus patients with perioperative cardiovascular complications (7.5 days, IQR 2.5-10). However, warfarin interruption ≤3 days before surgery was an independent predictor of bleeding complications.

## Discussion

The initial idea of our study was to see the results of this registry and then (if there proved to be some trend for less cardiovascular events in patients with continuing ASA treatment) consider a randomised study comparing the two strategies (a one-week ASA interruption vs. no interruption at all). As no trends were observed, we decided not to initiate a randomised study. In our current view (knowing the registry results) such a study would most likely be futile.

The independent predictors of in-hospital mortality (age, preoperative anaemia, history of chronic heart failure, acute surgery and general anaesthesia) are one of the important findings of this study. The relative importance of bleeding events versus cardiovascular events is reflected in the fact that the number of deaths related to cardiovascular complications was 34 vs. only 2 deaths related to bleeding. Perioperative cardiovascular complications are usually more serious and more difficult to treat than most bleeding complications [9, 10].

One study detected perioperative myocardial ischaemia in 40.5 % of patients. However, major adverse cardiac events occurred in only 8 % [11]. A large study in patients with atherosclerosis or at risk for it undergoing non-cardiac surgery found myocardial infarction in 5 % and troponin release in 8 % of patients [12]. In general surgical patients with documented coronary artery disease or at high risk for it, Filipovic et al. found troponin elevation in 16 % and myocardial ischaemia on continuous ECG in 46 % of patients [13].

The role and timing of interruption of antiplatelet agents before non-cardiac surgery has been studied by several authors. The STRATAGEM study randomised 291 patients undergoing *elective* non-cardiac surgery to either aspirin or placebo starting 10 days before surgery. The study did not find a significant difference in the number of major complications between the aspirin vs. placebo groups [14]. Aspirin increased the rate of bleeding complications in a meta-analysis by factor 1.5; however it did not lead to more severe bleeding complications with the exception of intracranial surgery and transurethral prostatectomy [15]. A single-centre study analysed 516 ASA-treated patients undergoing elective non-cardiac surgery: 289 patients had antiplatelet therapy stopped in the perioperative period. The decision to cease antiplatelet therapy, which occurred commonly, did not appear to be guided by perioperative cardiac risk stratification [16]. A Swedish study concluded that in high-risk patients undergoing non-cardiac surgery, perioperative aspirin reduced the risk of major adverse cardiac events without increasing bleeding complications [17].

The role of clopidogrel was investigated by Burdess et al.: in patients with critical limb ischaemia, perioperative dual antiplatelet therapy reduces biomarkers of atherothrombosis without causing unacceptable bleeding [18].

Our study seems to confirm that previous PCI with coronary stent implantation is an independent risk factor for perioperative complications. Savonitto et al. [19] and Motovska [20] recommend stratifying the risk of surgical bleeding and cardiac ischaemic events in order to manage perioperative antiplatelet therapy whenever surgery cannot be postponed.

The situation is even more difficult when patients are treated with long-term oral anticoagulation. The analysis of 1024 patients with warfarin therapy interruption before minor surgery found 7 post-procedure thromboembolic events and 23 clinically significant bleedings [21]. Another study on 556 patients with mechanical prosthetic valves evaluated the risk of thrombotic and bleeding complications when warfarin was stopped 4–5 days prior to a surgical procedure and re-started after the procedure as soon as haemostasis was assured. The 3-month incidence of thromboembolism was 0.9 %. None of those few cases were fatal. The cumulative incidence of major bleeding was 3.6 % and was fatal in 0.2 % [22]. Douketis et al. analysed 650 consecutive patients with a mechanical heart valve, chronic atrial fibrillation, or embolic stroke who required interruption of warfarin therapy because of an invasive procedure. Warfarin was stopped 5 or 6 days before the procedure, and patients received subcutaneous dalteparin starting 3 days before the procedure. Two patients died due to thromboembolism, 2 patients suffered non-fatal thromboembolic complications, there were 6 major bleeding episodes and 32 patients had episodes of increased wound-related blood loss [23].

The POISE-2 study (www.clinicaltrials.gov, identifier NCT01082874) is a large trial investigating the effectiveness of aspirin and clonidine versus placebo (factorial 2 × 2 design) in 10,000 patients with coronary artery disease undergoing non-cardiac surgery. The aim is to provide data to guide the decision-making on aspirin in the perioperative setting. The hope is that in the future perioperative antiplatelet therapy will be based on clinical evidence rather than on consensus.

### Our study limitations

This study was designed to include all-comers with various cardiovascular diseases undergoing major surgery with no exclusion criteria. Over one-third of the patients were admitted (and operated) acutely without sufficient time to withdraw the antithrombotic medication. Some of our findings may be influenced by this fact. The all-comers design is a strength of this study (giving a real-life picture) but simultaneously a weakness (causing heterogenicity of patients, their medications and surgical procedures). This study has no power to assess dual antiplatelet therapy in this setting or to produce any recommendations regarding patients with coronary stents or with prosthetic valves due to the relatively low number of these patients within the studied population.

As the study aim was to describe the real-life situation in nonselected consecutive patients, we did not prescribe any prospective criteria for when to stop the antithrombotic therapy. The number of patients on warfarin was 296 and thus the study was not powered to analyse this group. Very few patients were on the newer antithrombotic agents (dabigatran). The relatively low number of patients with perioperative complications is clinically encouraging, but statistically is certainly a limitation for the interpretation of the study results.

Another serious limitation for the interpretation of our study results is the fact that most patients received low-dose perioperative anticoagulation to prevent venous thromboembolism. This might further complicate the interpretation of our data.

## Conclusions

Perioperative cardiovascular complications in these high-risk elderly all-comer surgical patients with known cardiovascular disease are relatively rare, but once they occur, the case fatality is high. Perioperative bleeding complications are more frequent, but their case fatality is extremely low. Patterns of interruption of chronic aspirin therapy before major non-cardiac surgery are not predictive for perioperative complications (neither cardiovascular, nor bleeding). Simple baseline clinical factors are better predictors of outcomes than antithrombotic drug interruption patterns.
